# Primary hypothyroidism presenting with Torsades de pointes type tachycardia: a case report

**DOI:** 10.1186/1757-1626-1-298

**Published:** 2008-11-06

**Authors:** Mohammad Shojaie, Ahad Eshraghian

**Affiliations:** 1 Internal Medicine Department, Jahrom University of Medical Science, Motahari Boulevard, Jahrom, Fars Province, Iran; 2 Cardiology Department, Jahrom University of Medical Science, Motahari Boulevard, Jahrom, Fars Province, Iran

## Abstract

**Background:**

Hypothyroidism can manifest with cardiac abnormalities, often consisting of a combination of morphologic and functional changes. Low voltage, sinus bradycardia, and slowed conduction are usually found on electrocardiography.

There are few reports of occurrence of torsades de pointes as the first presentation of long QT syndrome in the course of hypothyroidism.

**Case presentation:**

In present report we briefly describe a 50-years-old woman with severe hypothyroidism who presented with presyncope, prolongation of the QT interval, and polymorphic ventricular tachycardia (torsades de pointes).

**Conclusion:**

Our patient responded well to treatment with levothyroxine and QT intervals normalized and ventricular tachycardia was abolished two months after levothyroxine therapy.

## Background

Hypothyroidism has various cardiovascular manifestations including impaired diastolic function, reduced contractility and infrequently pericardial effusion and heart failure. Electrocardiographic (ECG) changes in hypothyroidism are bradycardia, right bundle branch block (RBBB), flat or inverted T wave, QRS prolongation, QT prolongation and infrequently ventricular arrhythmia, torsadess de pointes. We describe a patient with severe hypothyroidism who presented with presyncope and polymorphic ventricular tachycardia (torsadess de pointes) and treated with levothyroxine.

## Case presentation

**Figure 1:**
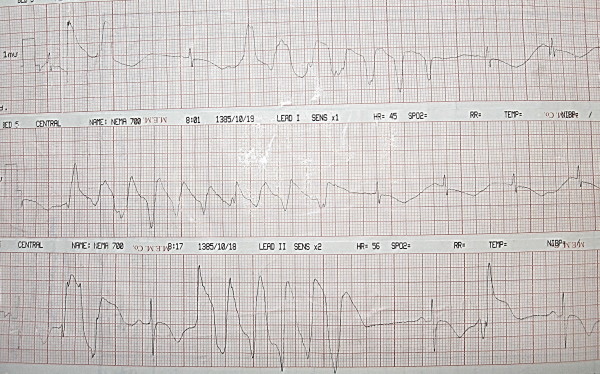
ECG showing Torsadess de pointes type tachycardia.

A 50-years-old woman was presented to the emergency department with chest pain and dyspnea. She was a case of single kidney. She had no history of systemic disease such as diabetes and hypertension. On the day of admission she collapsed and was unresponsive for a short while. She had not suffered from any episodes of syncope before. Physical examination revealed a well nourished woman with a blood pressure of 90/60 mmHg and a pulse rate of 100 beats per minutes. She had a puffy face and examination of the neck revealed no struma. The jugular venous pressure was normal. Cardiac auscultation was normal and the lungs were clear. Peripheral pulses of radial, femoral and dorsalis pedis were present. Electrocardiography (ECG) showed torsades de pointes type ventricular tachycardia (Fig 1). The patient received Magnesium and transferred to cardiac care unit (CCU).

**Figure 2:**
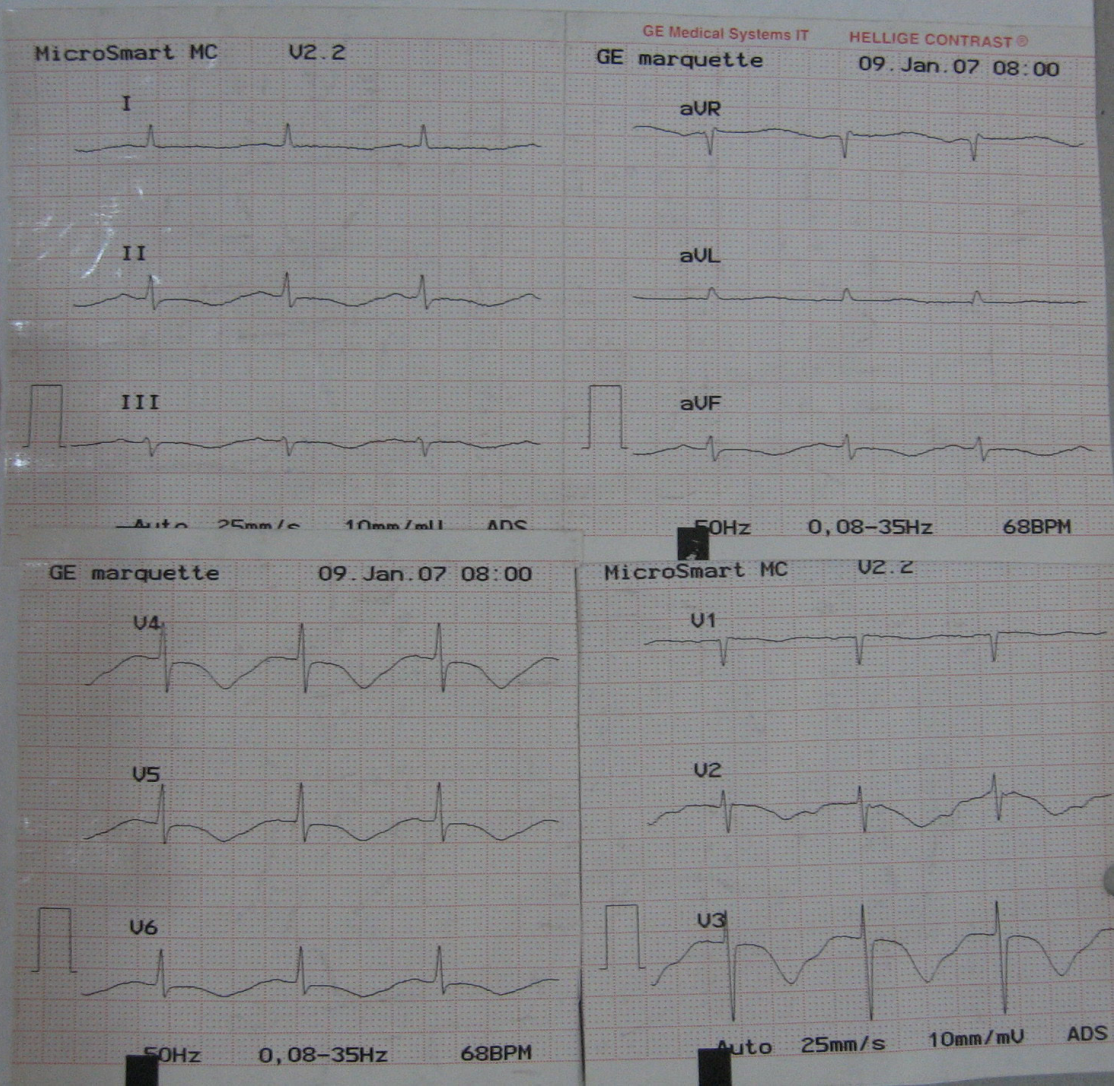
ECG showing long QT interval.

Next ECG obtained from the patient revealed T wave inversion and prolongation of QT intervals of 0.71 S (Fig 2). So the patient received phenytoin as treatment for prolongation of QT intervals. At the third day of admission the patient developed positional vertigo and her blood pressure dropped to 80 mmhg (pulse). Evaluation of thyroid function was recommended after consultation with neurologist. Thyroid function test revealed profound hypothyroidism. Total T_4 _was 0.71 μg/dL, free T_4 _(FT4) was 0.1 ng/mL, total T_3 _was 74 μg/dL and thyroid stimulating hormone (TSH) was 36 μU/mL. Other laboratory data such as blood urea nitrogen (BUN), Creatinine and electrolytes were in normal range. So the patient received levothyroxine 100 μg/day. Two months after treatment with levothyroxine, QT intervals normalized and ventricular tachycardia was abolished. Her periorbital edema had diminished and both TSH and free T4 had normalized.

## Discussion

Hypothyroidism results from reduced secretion of both T_3 _and T_4_, occurring in most cases as a consequence of destruction of the thyroid gland itself, usually by an inflammatory process. In some cases, it is secondary to decreased secretion of TSH, due to either pituitary or hypothalamic disease [1]. It is well known that an excess or deficit of thyroid hormones effect the cardiovascular system. The electrocardiographic changes in hypothyroidism include sinus bradycardia, low voltage QRS complexes and prolongation of QT interval. The P wave amplitude is usually very low and complete or incomplete right bundle branch block has been observed in patients with hypothyroidism. Cold intolerance, dryness of skin, weakness, constipation, hoarseness, shortness of breath, impairment of memory, menstrual dysfunction and even heart failure are common sign and symptoms of hypothyroidism. Cardiovascular manifestations of hypothyroidism include significant bradycardia, cardiac dilatation, weak arterial pulses, hypotension, distant heart sound, nonpitting facial and peripheral edema and evidence of congestive heart failure such as ascitis, orthopnea and paroxysmal dyspnea [2].

The physiological chronotropic response and normal tension of the heart muscle in diastolic phase depend on the proper expression of tri-iodothyronine in the heart cells and its stimulating influence on Na+-K+ ATPase and Ca2+ ATPase in endoplasmic reticulum. Normal heart contractility is also related to proper tri-iodothyronine-stimulated transcription of the myosin heavy-chain alpha gene and inhibition of the heavy-chain beta gene. Moreover, proper tri iodothyronine expression in the cardiac muscle affects the number of b-adrenergic receptors and their sensitivity to catecholamines.

Profound hypothyroidism and decreased expression of tri-iodothyronine in the heart cells may cause a worsening of cardiac contractility, a decreasing heart rate and a slowing down of the conduction of electrical stimuli in the heart muscle. This may be the reason for bradycardia and elongation of the QT interval and, in consequence, life-threatening arrhythmias may occur, for example torsades de pointes-type tachycardia.

The upper limit for duration of the normal QT interval corrected for heart rate (Q Tc) is often given as 0.44 sec. The term torsades pointes refer to a ventricular tachycardia characterized by QRS complexes of changing amplitude that appear twist around the isoelectric line. This term is usually used to describe a syndrome characterized by prolonged ventricular repolarization with QT intervals usully exceeding 500 msec [2].

There are few published reports of occurrence of torsades de pointes as the first presentation of long QT syndrome in the course of hypothyroidism. Hanslik et al. described a woman with myxoedema coma who initially presented a bizarre ECG with excessively prolonged QT intervals as predominant feature [3].

Kukala et al reported a 78 year-old woman with primary hypothyroidism and atrial fibrillation treated with sotalol, complicated with cardiac arrest due to ventricular fibrillation (VF) and torsades de pointes [4]. Chojnowski et al reported a 51-years-old woman with Hashimoto disease and hypothyroidism with repeated torsade de piontes tachycardia and cardiogenic sock in the course of her disease [5]. In two above mentioned cases the patients were known cases of hypothyroidism and torsades de piontes; although is not frequent, was occurred in the course of their diseases.

However, occurrence of torsades de pointes as the first manifestation of hypothyroidism is very rare. In 2006 Schenck et al. described a patient with severe hypothyroidism who presented with presyncope, prolongation of the QT interval, and polymorphic ventricular tachycardia (torsades de pointes) [6]. In 1983 Fredlund and Olsson reported two patients with long QT interval, ventricular tachycardias of torsades de pointes type and repeated ventricular fibrillation episodes, who also turned out to have significant hypothyroidism [7]. As expected, our patient responded well to treatment with levothyroxine and QT intervals normalized and ventricular tachycardia was abolished two months after levothyroxine therapy.

## Consent

Written informed consent was obtained from the patient for publication of this case report and accompanying images. A copy of the written consent is available for review by the Editor-in-Chief of this journal.

## Competing interests

The authors declare that they have no competing interests.

## Authors' contributions

MS managed the patient, analyzed and interpreted the patient data. AE analyzed and interpreted the patient data and was a major contributor in writing the manuscript.
